# Ribcage measurements indicate greater lung capacity in Neanderthals and Lower Pleistocene hominins compared to modern humans

**DOI:** 10.1038/s42003-018-0125-4

**Published:** 2018-08-16

**Authors:** Daniel García-Martínez, Nicole Torres-Tamayo, Isabel Torres-Sánchez, Francisco García-Río, Antonio Rosas, Markus Bastir

**Affiliations:** 1 Paleoanthropology Group, Museo Nacional de Ciencias Naturales (CSIC), José Gutiérrez Abascal 2, 28006 Madrid, Spain; 2 Hospital Universitario La Paz, Biomedical Research Institute (IdiPAZ), 28046 Madrid, Spain

## Abstract

Our most recent fossil relatives, the Neanderthals, had a large brain and a very heavy body compared to modern humans. This type of body requires high levels of energetic intake. While food (meat and fat consumption) is a source of energy, oxygen via respiration is also necessary for metabolism. We would therefore expect Neanderthals to have large respiratory capacities. Here we estimate the pulmonary capacities of Neanderthals, based on costal measurements and physiological data from a modern human comparative sample. The Kebara 2 male had a lung volume of about 9.04 l; Tabun C1, a female individual, a lung volume of 5.85 l; and a Neanderthal from the El Sidrón site, a lung volume of 9.03 l. These volumes are approximately 20% greater than the corresponding volumes of modern humans of the same body size and sex. These results show that the Neanderthal body was highly sensitive to energy supply.

## Abstract (web summary)

Daniel García-Martínez et al. report Neanderthal lung volume estimates based on measurements from rib bone fossils and lung capacity data from modern humans. They estimate that Neanderthal individuals had approximately 20% higher lung capacity than modern humans, possibly due to higher energy requirements.

## Introduction

Neanderthals are heavy-bodied hominins with relatively short distal limbs and wide trunks^1–9^ consisting of a wide pelvis^10–13^ and a wide central–lower thorax^14–20^. Therefore, their body shape was characterized as “short but massive”^9^, with similar proportions to current cold-adapted populations^2^ but heavier and more muscular. They also showed larger brains than modern humans did in absolute terms, presenting an average cranial capacity of around 1600 cc for males and 1300 cc for females^21–26^. It has recently been proposed that, even though the Neanderthal cerebellum was smaller than in early *Homo sapiens*, the differences in Neanderthal cerebrum size compared to modern humans are the result of the larger occipital lobes^27^.

The evolutionary origin for the Neanderthal body shape in the European hominin lineage can be certainly found in the Sima de Los Huesos site (Burgos, Spain)^6,28,29^. According to these studies, the short but massive body shape found in Neanderthals was inherited from their Middle Pleistocene ancestors *H. heidelbergensis*, even though they were probably slightly taller than Neanderthals^6,28,29^. Some authors have also proposed wide trunks, citing fossil evidence of the European Lower Pleistocene from the Gran Dolina site ATD6^6,30,31^. However, this morphological evidence should be interpreted with some caution, because the fossil record at the Gran Dolina site is more scarce and fragmentary than that of the Sima de Los Huesos site^6^. In addition, we must be cautious about the evolutionary significance of the fact that wide and heavy bodies are found in both *H. antecessor* and Neanderthals: although some authors have proposed *H. antecessor* as an ancestor of both modern humans and Neanderthals^32,33^, this is only one of other potential evolutionary scenarios^34^.

Regardless of whether the short and massive bodies of Neanderthals evolved in the European Middle or Lower Pleistocene, there is agreement about their wide pelvises and central–lower ribcages. A potential explanation for the wide trunk of Neanderthals is based on bioenergetics^9,35,36^. Neanderthals should have shown greater oxygen consumption than modern humans not only in order to maintain the basic metabolism of a heavier body but also in order to provide oxygen to their large brain and muscles, which require large amounts of oxygen^9,35,37^. Using estimates of daily energetic expenditure (DEE) in Neanderthals and current human populations, Froehle and Churchill^36^ found that Neanderthals tended to expend more energy than their modern human counterparts under all climatic conditions (cold, temperate and warm) and in both sexes. However, how would Neanderthals have obtained the large amount of oxygen needed to maintain their high DEE? They would have done so with a large and powerful ribcage, which could explain (at least partially) the wide trunks of Neanderthals.

There is agreement that the Neanderthal thorax was relatively larger than ours in the central–caudal area (but see Chapman et al.^38^), but it is less clear whether the Neanderthal thorax was large for their mass and stature^35^. In addition, the Neanderthal thorax was mediolaterally expanded in the central–caudal area compared to that of modern humans^15,17–20^. From a functional point of view, it is important to note that the central–lower thorax (ribs 7–12) is where the diaphragm, one of the main muscles responsible for respiration, is attached^39–41^. Since this area of the ribcage is mediolaterally broader and large in Neanderthals, authors have proposed a larger diaphragmatic surface linked to greater diaphragmatic power and excursion during breathing cycles compared to modern humans^14–20^. In addition, it has recently been observed that the lower thorax contributes to kinematic thorax size changes more than the upper thorax does, so evolutionary changes in the caudal area would have had a greater functional impact than changes in the upper area^42^.

These interspecific functional differences in ribcages, probably caused by the greater need for oxygen intake in Neanderthals, should be reflected in their respiratory parameters. Total lung capacity (TLC), understood as the maximum amount of air that lungs can hold in maximum inspiration after a maximal expiration^43^, has been used to address differences in oxygen intake in modern humans^44^. Specifically, these authors used maximum anteroposterior and mediolateral thoracic diameters measured in X-ray images to study their correlation with TLC. However, estimating TLC in fossil individuals becomes harder, since there are only isolated elements in the fossil record, and regressions calculated for mediolateral and anteroposterior diameters in anatomically connected thoraces cannot be used. Therefore, TLC estimates for Neanderthals, based on measurements of individual elements of the ribcage, are necessary and have not been performed to date.

The aim of this paper is to fill this gap of knowledge using traditional measurements and three-dimensional (3D) geometric morphometrics of individual ribs of healthy volunteers whose TLC is known in order to calculate regressions of individual rib size on TLC. We use our regressions to estimate the TLC for a female (Tabun 1) and a male (Kebara 2) Neanderthal, using their best-preserved ribs in order to measure their costal size. We also estimate the TLC for another Neanderthal specimen of unknown sex from the El Sidrón site as well as for ATD6 hominins, in light of the scenario which hypothesized that the large TLC for Neanderthals was inherited from their possible ancestors of the Lower Pleistocene in Europe. Finally, we explore whether these species presented large TLC values for their stature (TLC/S ratio) and body mass (TLC/M ratio).

## Results

### Overview

**Fig. 1:**
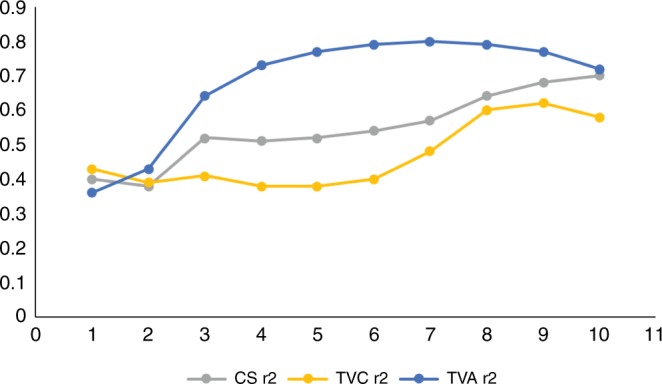
Variation along the costal sequence (1–10) of *r*^2^ of linear regressions for centroid size (CS; grey) and tubercle-ventral chord (TVC; yellow) on TLC, as well as exponential regression of tubercle-ventral arch (TVA_sml; azul) on TLC. For every rib, except for ribs 1, correlations were greater using TVA_sml than using TVC or CS

Raw values of tubercle–ventral arch, calculated as the cumulative distance between semilandmarks (TVA_sml; see Methods) of individual ribs from the modern human sample, as well as TLC, stature and lean body mass associated with the individuals they come from, can be observed in Supplementary Data 1 and 2. Raw values of TVA_sml and estimated stature and lean body mass for fossil specimens can also be observed in Supplementary Data 1 and 2. Results of exponential regression analysis, by level, of rib size on TLC, can be observed in Table 1 and Fig. 1. Even though all the regressions are statistically significant (permutation test; *p* < 0.0003), the rib TVA_sml was more correlated with TLC in central–caudal ribs. When we studied the correlation of tubercle–ventral chord (TVC) and centroid size (CS) with TLC (linear regressions), we found smaller correlations at every rib level except for the first level, than comparing TVA_sml vs. TLC correlations (Fig. 1). Therefore, we decided to use the TVA_sml approach instead of TVC or CS to calculate TLC. Despite those lower correlations, the formulae of linear regressions for calculation of TLC using TVC and CS are given in Supplementary Tables 1 and 2.

**Table 1:** Results of exponential regression analysis of TVA_sml by level on TLC

	r^2^	Formula	*p* Value
1st	0.36	*y* = 1.6158e^0.0115×^	0.0002
2nd	0.43	*y* = 0.8882e^0.0095×^	0.0002
3rd	0.64	*y* = 0.3785e^0.0112×^	0.0001
4th	0.73	*y* = 0.3077e^0.0109×^	0.0001
5th	0.77	*y* = 0.315e^0.0103×^	0.0001
6th	0.79	*y* = 0.3329e^0.01×^	0.0001
7th	0.80	*y* = 0.3218e^0.0103×^	0.0001
8th	0.79	*y* = 0.3131e^0.0109×^	0.0001
9th	0.77	*y* = 0.427e^0.0107×^	0.0001
10th	0.72	*y* = 0.4536e^0.0121×^	0.0001

*r*^2^, formulae and statistical significance of the regressions are shown

### Absolute TLC in Neanderthals and Lower Pleistocene hominins

We used ribs 5 and 7 left and 10 right from the Kebara 2 individual; ribs 6–8 left from the Tabun 1 individual; rib 5 right from the El Sidrón site^20^ and ribs 7 (ADT6–89+206) and 10 left (ADT6–39) from ATD6 hominins to calculate their TLC. These ribs were the only ribs available in the record that could be measured following our measurement protocol (TVC, TVA_sml and CS). Estimations of TLC in fossil specimens using TVA_sml regressions yielded, in the Kebara 2 male, values of 6.42 l, 11.34 l and 9.37 l for ribs 5, 7 and 10, respectively (mean 9.04 l); for the Tabun 1 individual, values of 5.71, 6.03 l and 5.80 l for ribs 6–8, respectively (mean 5.85 l); a value of 9.03 l for the El Sidrón individual and for the ATD6 hominins, values of 5.28 l and 8.70 l for ribs 7 and 10, respectively. The mean TLC value for our male comparative sample was 7.20 l (95% confidence interval (CI): 6.80–7.59) and the mean TLC value for our female comparative sample was 4.85 l (95% CI: 4.62–5.01). Therefore, TLC estimations of the Kebara 2 and Tabun 1 individuals were (on average) larger than the mean of their corresponding modern human samples and also outside the 95% CI, and estimation of TLC using the rib 5 from the El Sidrón site yielded a value (9.03 l) that was outside the corresponding CI for male modern humans. Estimation of TLC of ATD6 using rib 7 was larger than the female average and outside the 95% CI of females, and TLC estimation using rib 10 was larger than the male average and outside the 95% CI of males. TLC estimations and statistics for our modern human comparative sample can be observed in Table 2.

**Table 2:** Mean, standard deviation (sd.) and 95% confidence interval (95% CI) of total lung capacity (TLC), as well as TLC relative to stature (TLC/S) and lean body mass (TLC/M) for males and females of our comparative sample of modern humans

	Male mean, sd.	95% CI	Female mean, sd.	95% CI
TLC (l)	7.20, 0.77	6.80–7.59	4.85, 0.41	4.62–5.01
TLC/S	0.041, 0.004	0.039–0.044	0.030, 0.004	0.029–0.031
TLC/M	0.125, 0.021	0.114–0.136	0.108, 0.019	0.098–0.117

### TLC in Neanderthals and Lower Pleistocene hominins relative to stature and body mass

**Fig. 2:**
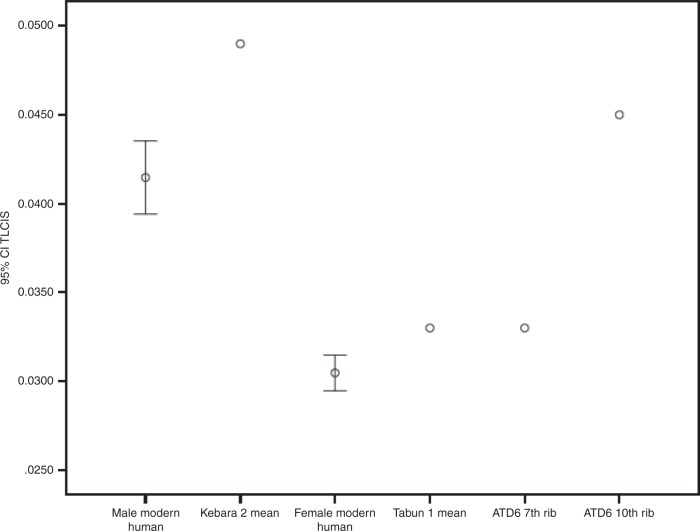
Bivariate plot showing TLC relation with stature, observing the 95% confidence intervals for modern humans as well as fossil values where stature was known in the literature

Estimations of TLC/S ratio yielded, in the Kebara 2 individual, values of 0.039, 0.068 and 0.056 for ribs 5, 7 and 10, respectively (mean 0.054). For the Tabun 1 individual, values of 0.037, 0.039 and 0.037 were calculated for ribs 6–8, respectively (mean 0.038). For ATD6 hominins, values of 0.031 and 0.050 were calculated for ribs 7 and 10, respectively. The mean value of TLC/S for our male comparative sample was 0.041 (95% CI: 0.039–0.044) and the mean value for our female comparative sample was 0.030 (95% CI: 0.029–0.031). Therefore, TLC/S estimates of the Kebara 2 and Tabun 1 individuals were (on average) statistically larger than their corresponding modern human samples (Fig. 2). The ATD6 TLC/S ratio, estimated using rib 7, was larger than the female average but at the upper limit of the 95% CI, whereas when estimated using rib 10, it was larger than the male 95% CI (Fig. 2). In the El Sidrón rib, TLC/S could not be calculated since its stature was not available. TLC/S statistics for our modern human sample can be observed in Table 2.

Finally, estimations of TLC/M ratio yielded, in the Kebara 2 individual, values of 0.099, 0.174 and 0.144 for the ribs 5, 7 and 10, respectively (mean 0.139). For the Tabun 1 individual, values of 0.120, 0.127 and 0.122 were calculated for ribs 6–8, respectively (mean 0.123). This value could not be calculated for the El Sidrón individual or the ATD6 hominins, because their body mass values are not available in the current literature. The mean value of TLC/M for our male comparative sample was 0.125 (95% CI: 0.114–0.136) and the mean value for our female comparative sample was 0.107 (95% CI: 0.098–0.117). TLC/M estimations for Kebara 2 and Tabun 1 were (on average) larger than their corresponding modern human sample, being outside their 95% CI. TLC/M statistics for our modern human comparative sample can be observed in Table 2.

## Discussion

TLC as an absolute measurement related to respiratory volume can be used to address the issue of respiratory and energetic demands in modern humans^44,45^ and, potentially, in fossil hominins as well^9,35^.

However, although TLC values are obtained through a straightforward technique in hospital subjects (as in Bellemare’s^44^ study), it is more challenging when we deal with the fossil record. This is because we can only infer TLC from variables measured in individual elements of the ribcage such as the ribs and vertebrae. In this regard, our results are pioneering in showing that individual rib size (assessed throughout TVA_sml, TVC and CS) can be correlated with TLC. We also specify that, although our 3D measurement (CS) is more correlated with TLC than the traditional measurement TVC for ribs 3–10, the tubercle–ventral arch (TVA_sml) is even more informative about TLC. This is possibly caused by the fact that TVA captures information about mediolateral width and lung circumference, while TVC only captures anteroposterior size, which must influence also CS. In addition, we specify that the size of central–lower ribs is more correlated with TLC than is the size of upper ribs (Fig. 1). This is consistent with recent research which shows that lower thorax size is more correlated with functional size, understood as the size increase from maximum expiration to maximum inspiration^42^.

Our results for Neanderthals show that TLC presents absolute values that are larger than in their corresponding human counterparts (Table 3). Kebara 2, a male Neanderthal from Israel, shows a mean value of 9.04 l of TLC, which is statistically larger than our male human sample (mean = 7.20 l) and the one from Bellemare et al.^44^ (mean = 6.27 l). Our estimates for Tabun 1, a female Neanderthal from Israel, yielded a mean value of 5.85 l, which is statistically larger than our female sample (mean = 4.85 l) and the mean of Bellemare et al.^44^ (mean = 4.81 l). It should be noted that the male Kebara 2 TLC was 54% larger than the value for the female Tabun 1. The fact that this percentage is slightly larger in Neanderthals than in our modern human sample (around 48%, see above) could be the result of differences in body composition, because Kebara presents a larger lean mass compared to Tabun 1 than our male modern humans compared to our modern females (Table 3). The El Sidrón SD-1450 rib also provides insights into the Neanderthal TLC, and since it is statistically larger than our male sample, it is likely that it would have belonged to a male individual.

**Table 3:** Total lung capacity (TLC) values and associated variables (cranial capacity, lean body mass and stature) of the groups studied here

	Neanderthal male	Neanderthal female	Modern human male	Modern human female
Total lung capacity (l)	9.04	5.85	7.20	4.85
Cranial capacity (cc)	1600	1300	1400	1250
Lean body mass (kg)	65.02	47.70	58.44	45.72
Stature (cm)	166.00	156.00	173.35	158.11

TLC obtained from our results, cranial capacity is obtained from Aiello and Dean^21^, lean body mass and stature of modern humans are obtained from our results and those values of Neanderthals are obtained from Churchill^9^

It is important to note that, if we had tried to estimate TLC values of these fossil specimens using other variables (such as stature) from standard human equations, Tabun 1 TLC would have been estimated at 4.67 l, 4.50 l and 4.91 l using the formulae from Crappo et al.^46^, Roca et al.^47^ and Quanjer et al.^48^, respectively. Had we used standard human equations to estimate the Kebara 2 TLC value, we would have obtained values of 6.18 l, 6.20 l and 6.36 l using the formulae from Quanjer et al.^48^, Cordero et al.^49^ and Neder et al.^50^, respectively. Therefore, both Kebara 2 and Tabun 1 present much larger values of TLC using our equations than when using human standard equations. Because different equations are used depending on the sex, we did not calculate this value for the El Sidrón Neanderthal and ATD6 hominins, since their sex was not known.

**Fig. 3:**
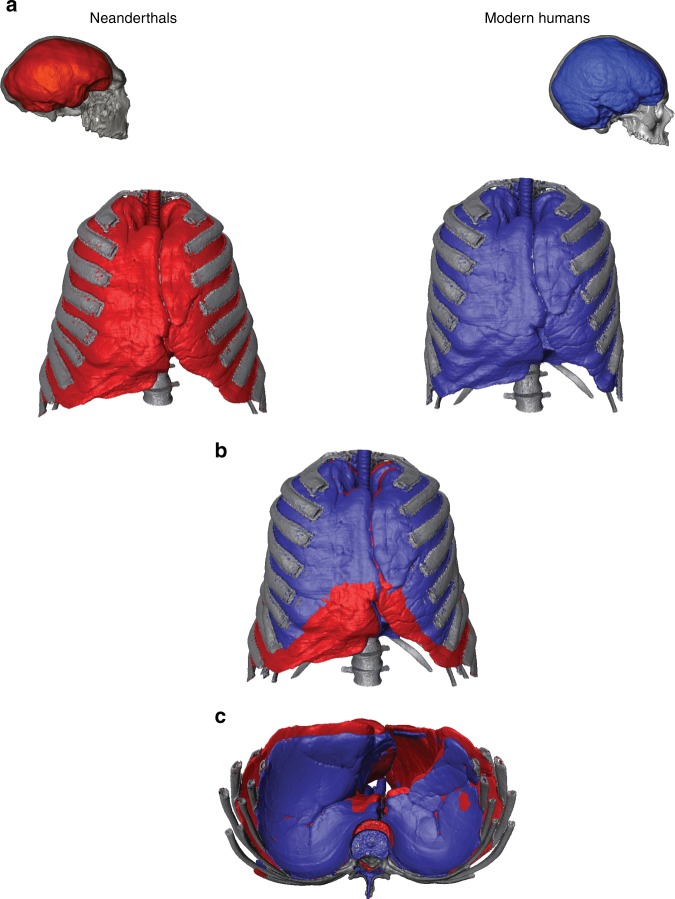
**a** Thorax and lungs' shape in the frontal view in modern humans and Neanderthals and their associated brains in the lateral view. Neanderthal thorax and skull belong to Kebara 2^5^ and Guattari Neanderthals, respectively. Modern human thorax and skull belong to an average of four modern humans^82^ and OI-2053, respectively. **b** Superimposition in the frontal view of the Neanderthal and modern human ribcages. **c** Superimposition in the caudal view of the Neanderthal and modern human ribcages

Recent evidence suggests that the large TLC observed in Neanderthals compared to modern humans was the result of large ribs in the central–lower thorax coupled with a more dorsal orientation of the transverse processes in Neanderthals compared to modern humans, causing mediolateral expansion of the ribcage^18,20^. This ribcage morphology (Fig. 3), combined with our results of TLC for Neanderthals, is consistent with a large oxygen intake to maintain their expected high DEE proposed by previous authors^9,35,51^. That large DEE must be caused for their large brains (Fig. 3, Table 3) and large lean body mass (Table 3), but alternative explanations such as the possibility that Neanderthals had large guts (liver and urinary systems) necessary for processing large amounts of meat, could also be linked with high DEE^52^.

Even though there is agreement about the large size of the Neanderthal ribcage^14–20^, it is not as clear whether their ribcages were larger for their body mass or their stature^35^. For example, estimates of the Shanidar 3 Neanderthal respiratory area of rib 8 suggest that it was proportional to his body mass but that the respiratory area of the Kebara 2 rib 8 was relatively larger for his body mass^35^. In this regard, our results could suggest that both Kebara 2 and Tabun 1 presented a larger TLC/M ratio than our modern human reference samples, which supports Churchill’s^35^ work. As for whether Neanderthals present larger TLC for their stature compared to modern humans, our results support this assertion since TLC/S values of Kebara 2 and Tabun 1 individuals were (on average) larger than their corresponding modern human samples. The fact that both Neanderthals presented larger TLC/M and TLC/S values compared to our modern human sample must be related to their large DEE.

However, some caution must be taken in the interpretation of these results since TLC/M and TLC/S ratio are based on estimates of stature and lean body mass in Neanderthals^9,35,51,53^ and this could introduce some error in the ratios. Even when including this potential error, it is clear that Neanderthals’ thoraces were larger for their stature (Fig. 2), which would be consistent with previous research on ribcage/body size ratios based on rib size/humerus length^15^. It is also important to recall that lean body mass estimates were calculated applying fat-free mass percentages to the total Neanderthal body mass, which were taken from modern Inuit individuals^51,54,55^. Therefore, it is possible that the percentages for Neanderthals were different than those of Inuits. In addition to differences in fat-free mass percentages, there may also be differences in other tissues, such as brown adipose tissue. Although the role of this tissue in environmental adaptation is speculative, it is the only human tissue dedicated exclusively to heat production^56^. Body composition in Neanderthals is not the focus of our work and should be addressed in future research.

Regarding the evolutionary origin of the large Neanderthal TLC, *H. heidelbergensis* (likely potential ancestors of Neanderthals) are also thought to have large thoraces, both in absolute terms and perhaps relative to their stature as well^6,57^. However, the lack of literature on fossil remains of the costal skeleton makes it difficult to address this issue. Lower Pleistocene hominins from the Gran Dolina site (Burgos, Spain) are hypothetical ancestors of *H. heidelbergensis* (and thus Neanderthals) and are also thought to have large thoraces because of their long clavicles^6,30^. Whether *H. antecessor* is actually a species itself or represents an European branch of *H. erectus*/*ergaster*^34^, recent Bayesian analyses^58,59^ suggest that *H. antecessor* belongs to a basal clade of modern human and Neanderthals, alongside other early *Homo* species such as *H. erectus*, *ergaster* and the recently discovered species named as *H. naledi*^60^. Therefore, *H. antecessor* could be used as an approach to test whether large bodied early *Homo* species already presented a large TLC.

Our results of estimated TLC based on ribs 7 and 10 yielded values of 5.28 l and 8.70 l for ATD6 hominins, respectively, which were larger than our comparative sample of female and male modern humans, respectively. In this case, we are not certain that these ribs belonged to the same individual, so we hypothesize here that ATD6–39 (the larger value) could represent a male rib, whereas ATD6–89+206 (the smaller value) could represent a female rib. If this is confirmed, we would see in ATD6 hominins the same evolutionary trend that we see in Neanderthals, males and females being larger (on average) than our modern human comparative sample. However, some caution should be taken because of the uncertainty in the composition of the ATD6 sample^31^. The TLC/M ratio for these hominins could not be calculated since body mass values are not available in the current literature due to the fact that this fossil site did not yield any remains of lower limbs that were well enough preserved to provide evidence of body mass^30^. Regarding stature, ATD6 hominins presented an average stature of 172.5 cm, which was larger than the average for Neanderthals^6,30,57^. The TLC/S ratio for ATD6 hominins using rib 7 was larger than the female average and larger than the male average using rib 10. This would support the possibility that ribs ATD6–89+206 and ATD6–39 are female and male ribs, respectively. It would also support what we found in Neanderthals, that is, that the large TLC relative to stature was beginning to be evident in the Lower Pleistocene of Europe, even when considering that ATD6 hominins presented larger statures than Neanderthals^6,30^.

Therefore, according to the evidence of TLC, if we accept that *H. antecessor* was in the basal clade of both Neanderthals and modern humans, we suggest here that a large ribcage relative to stature is present in the whole European hominin lineage (represented here by ATD6 hominins and Neanderthals). However, whether it is also present in other European hypothetically intermediate species such as *H. heidelbergensis* must be addressed in future research. The large ribcage of the European hominin lineage could be linked to the wide trunks proposed by previous authors for those species^6^, which would show an evolutionary trend towards Neanderthals, based on relative stature reduction and relative thorax size increase.

Regarding the adaptive significance of this evolutionary trend, it should be noted that in the Lower and Middle Pleistocene there is a trend towards large body sizes across most of the mammal clade, with herbivores showing a larger size increase than carnivores^61–65^. In carnivores, this size increase could be important for facilitating hunting tasks, whereas in herbivores it could be important for avoiding being preyed upon by carnivores. This general ecological rule could also apply to hominins and perhaps underlie the large body mass of Lower and Middle Pleistocene hominins, partially explaining their wide trunks^9^. Besides this general explanation, other more specific ones have been proposed: the stout (“short but massive”) Neanderthal body could be explained by the eco-geographical rules of Allen and Bergmann^66,67^, which could cause the shortening of distal limbs and the widening of the trunk observed in Neanderthals^1–9^. However, recent studies on bioenergetics show that Neanderthals inhabiting the same climatic conditions as modern humans present larger DEE than modern humans. This could be the result of the cost of maintaining heavy and highly muscled bodies with large brains (Fig. 3, Table 3) along with the need to exert muscular force in the accomplishment of subsistence tasks^9,35,36,51^. This larger muscle mass would have provided them with a greater thermogenic capacity and also greater insulation against cold compared to modern humans, which could be understood as an exaptation^9,35^. Future studies should include more Neanderthal ribs and also other hominin species not included here, such as *H. heidelbergensis* or *H. erectus*, in order to expand the evolutionary framework.

Finally, even though physiological function must have been of evolutionary significance, caution should be used in assuming that an enlarged thorax was result of natural selection and was passed down as an adaptation to later European Pleistocene hominins. In particular, enhanced pulmonary function as modelled in modern human populations living at high altitudes shows that developmental processes have an important role in shaping the physiology of respiration and oxygen consumption^68–72^. Developmental factors also play an important role in determining thorax morphology. Here again, humans living at high altitudes from many different regions provide important data demonstrating this point, but the small sample sizes of hominin fossil assemblages make developmental factors difficult to test. The possibility that developmental processes contributed to the emergence of a large thorax and pulmonary capacity in early Pleistocene hominins of Europe and in later Neanderthals does not alter the results of this study.

Our work is, to our knowledge, the first successful attempt to estimate TLC in fossil hominins. We have found that Neanderthals presented around 20% larger lung capacities than modern humans, both absolutely and relative to their lean mass and stature. This could be caused by the large lean body mass of Neanderthals, coupled with their large brains and gut size (liver and urinary systems), contributing to their high DEE. Assuming that *H. antecessor* is in the basal clade of Neanderthals (which is still a heated debate), the trend towards large lung capacities could even be observed in the lower Pleistocene of ATD6. Finally, although we used a large sample of current Europeans to create a statistical model (controlled for stature and body mass) to calculate TLC in fossil hominins, future research should include broader samples from different modern humans populations. Those that present different limb proportions compared to Europeans and that could parallel Neanderthal body proportions (populations adapted to high altitudes and extreme low temperatures) are mostly necessary. In addition, future studies should make an effort to include early *H. sapiens* such as Cro-Magnon, Skhul or Abri Pataud.

## Methods

### Material used

We used computed tomography (CT) reconstructions of rib cages that belonged to 36 adult Spanish individuals (17 males and 19 females) who were CT-scanned in maximum inspiration. The data were obtained from hospital subjects who were previously scanned as a healthy control group to be compared with pathological individuals belonging to a different research project at the Hospital Universitario La Paz (Madrid, Spain). In none of the cases could any pathologies affecting skeletal thorax form be observed. The sex composition is detailed in Supplementary Data 1. Consent was given to use these CT data for research and prior to the analyses all CT data were anonymized to comply with the Helsinki declaration^73^.

Fossil specimens used in this study comprise 3D surface scans of original costal remains from the Neanderthal male Kebara 2 (Mount Carmel, Israel), the Neanderthal female Tabun 1 (Mount Carmel, Israel) and the virtual reconstruction of the Neanderthal rib 5 from El Sidrón site SD-1450 (Asturias, Spain), as well as from high-quality casts of ribs from ATD6 hominins from the Atapuerca site (Burgos, Spain). Only the best-preserved fossils, where the rib shaft was complete from the articular tubercle to the distal end, were studied; this constrained the sample but prevented uncertainty that could be caused by estimates of missing data. Since recent studies have found that variation in lower ribs has a larger impact on functional dynamics than does variation in upper ribs^42^, in fossil specimens we only studied ribs from the central–lower thorax (from rib 5 onwards). Therefore, ribs 5 and 7 left and 10 right from the Kebara 2 individual; ribs 6–8 left from the Tabun 1 individual; rib 5 right from the El Sidrón site (virtual reconstruction of SD-1450^20^) and ribs 7 (ADT6–89+206) and 10 left (ADT6–39) from ATD6^31^ hominins were studied.

### Measurement of TLC and anthropometric variables and 3D data acquisition and quantification

For the comparative human sample, TLC was measured in litres (l) for each individual through spirometry using standard medical techniques. In order to study TLC in relation to stature, we measured stature in our modern human sample in standardized upright standing position using standard anthropometric techniques. For stature estimations of fossils, we used data from Froehle and Churchill^36^ for Neanderthals and from Carretero et al.^30^ for ATD6 hominins. In order to study TLC in relation to mass, we measured kilograms (kg) of fat-free mass (also called lean body mass) through bioimpedance using standard medical techniques in our comparative sample. For Neanderthals, only total body mass estimates were available^9,53^. In order to calculate lean body mass for Neanderthals Kebara 2 and Tabun 1, we used fat-free mass percentages from current Inuits, which have been successfully used by previous authors as a proxy for calculating this variable in Neanderthals^51,54,55^.

**Fig. 4:**
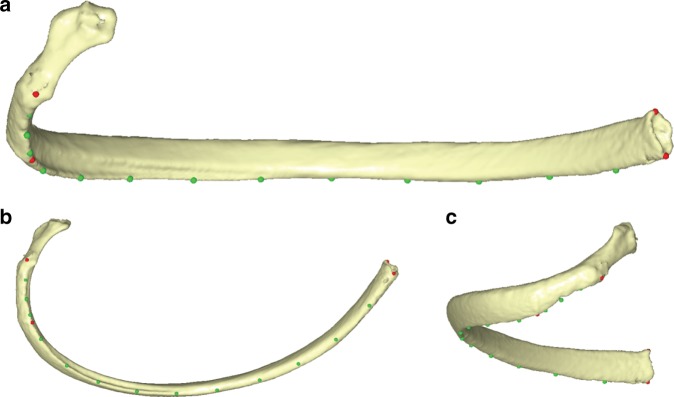
Landmarks and semilandmarks' digitization protocol. On the ribs, landmarks were placed at the most lateral point of the articular tubercle, the most inferior point at the *angulus costae* at the lower rib border (where the angle is most doubtlessly recognizable) and the most superior and inferior points of the sternal end. In addition, 13 equidistant semilandmarks were located along the lower costal border between the articular tubercle and the inferior sternal end

Each thorax of those individuals of which we measured TLC, lean body mass and stature was CT-scanned and segmented through a semiautomatic segmentation protocol of DICOM images using the software Mimics 8.0 (http://biomedical.materialise.com/mimics). In order to reduce the possible error related to left–right laterality, only ribs 1–10 from the left side were segmented from each thorax. The post-processing of the 3D surface models of skeletal elements (cleaning, smoothing and mesh hole-filling) was carried out by the Artec Studio v12 software (www.Artec3D.com) and the final 3D costal models were imported into the Viewbox4 software (www.dhal.com) for digitization. Then landmarks and semilandmarks for sliding^74–76^ were located on rib models following previous published protocols from García-Martínez et al.^77^. Since some fossil specimens (Tabun 1, Kebara 2 and ATD6 hominins) did not preserve the rib head, the three landmarks which described that structure in the comparative costal sample were excluded. The rib morphology was therefore described by 17 homologous 3D landmarks and sliding semilandmarks on each rib 1–10 (Fig. 4).

Once the landmarks were digitized, size data of costal elements were obtained as follows: (1) the TVC was calculated as the distance between the landmark at the tubercle and the landmark at the inferior point of the distal end. (2) The TVA_sml was calculated as the cumulative distance between the semilandmarks from the landmark at the tubercle and the landmark at the inferior point of the distal end. We used the term TVA_sml instead of TVA because our measurements do not exactly fit the definition of TVA from previous authors^15,78^. (3) Centroid size was obtained by Generalized Procrustes Analysis of the whole landmark data set^79–81^. Then we carried out regression analysis of the size of costal level on TLC in the comparative sample in order to explore what rib level size was most correlated to TLC. We observed that TLC on CS and TLC on TVC had a linear distribution, whereas TLC on TVA_sml had an exponential distribution. After that, we used these regressions to estimate TLC in fossil specimens using their size values. We explored the relationship between TLC estimates in the fossil specimens and the 95% CIs for the mean of the comparative sample. In addition, we also calculated TLC in relation to stature (TLC/S) and TLC in relation to lean body mass (TLC/M).

We also included a validation study, addressing the accuracy of the estimates using the different costal levels. For this aim, we estimated the TLC from the individuals of the comparative sample, which were already known, using the exponential regressions of TLC on TVA_sml that we obtained. We calculated, for every individual, the difference between the original known value and the estimate, using different rib levels. We observed that the best estimates (difference from the original of −0.01 l on average) were obtained using the seventh rib. This information can be found Supplementary Table 3 and Supplementary Figure 1.

### Data availability

The data sets generated during and/or analysed during the current study, besides that given in Supplementary Data 1 and 2, are available from the corresponding author on reasonable request.

## Electronic supplementary material

Supplementary Information

Description of Additional Supplementary Information

Supplementary Data 1

Supplementary Data 2
