# Supplementary material for: Ribcage measurements indicate greater lung capacity in Neanderthals and Lower Pleistocene hominins compared to modern humans
Source: Commun Biol. 2018 Aug 16;1:117. doi: 10.1038/s42003-018-0125-4 (PMC6123625; doi:10.1038/s42003-018-0125-4)

Supplementary online material

Supplementary table 1: Formulae for calculating TLC ("response" variable) using the TVC ("predictor" variable) of the ribs for every level.

	r^2	Formula
**Rib 1**	0.43	y = 0.1101x - 4.0695
**Rib 2**	0.39	y = 0.0773x - 4.6329
**Rib 3**	0.41	y = 0.0631x - 4.6863
**Rib 4**	0.38	y = 0.0537x - 4.4098
**Rib 5**	0.38	y = 0.0472x - 3.8866
**Rib 6**	0.4	y = 0.0459x - 4.1091
**Rib 7**	0.48	y = 0.0549x - 6.4793
**Rib 8**	0.6	y = 0.0675x - 8.8636
**Rib 9**	0.62	y = 0.0707x - 8.4055
**Rib 10**	0.58	y = 0.0692x - 6.5637

Supplementary table 2: Formulae for calculating TLC ("response" variable) using the CS ("predictor" variable) of the ribs for every level.

	r^2	Formula
**Rib 1**	0.4	y = 0.0666x - 2.8713
**Rib 2**	0.38	y = 0.0484x - 4.9768
**Rib 3**	0.52	y = 0.0502x - 8.1554
**Rib 4**	0.51	y = 0.0441x - 8.0715
**Rib 5**	0.52	y = 0.0389x - 7.313
**Rib 6**	0.54	y = 0.0374x - 7.1943
**Rib 7**	0.57	y = 0.0393x - 7.8643
**Rib 8**	0.64	y = 0.045x - 9.2277
**Rib 9**	0.68	y = 0.0488x - 9.0174
**Rib 10**	0.7	y = 0.0558x - 8.8182

Supplementary table 3: results from the validation study, showing the difference between the original TLC (l) and the estimations in the 36 individuals from our comparative sample, using different rib levels. Average and 95% confidence interval for this value are shown. As observed, the better results are produced using ribs 6 and 7.

	**1st ribs**	**2nd ribs**	**3rd ribs**	**4th ribs**	**5th ribs**	**6th ribs**	**7th ribs**	**8th ribs**	**9th ribs**	**10th ribs**
**Average**	-0.06	0.13	0.04	0.04	0.04	-0.02	-0.01	0.07	0.09	0.06
**-95% CI**	-0.42	-0.22	-0.24	-0.20	-0.19	-0.24	-0.20	-0.15	-0.13	-0.17
**+95% CI**	0.29	0.47	0.32	0.29	0.28	0.20	0.18	0.28	0.31	0.30

Supplementary Figure 1: Error bar graph showing the difference between the original known values and the estimates, using different rib levels. As observed, the better estimates are produced using ribs 6-7.

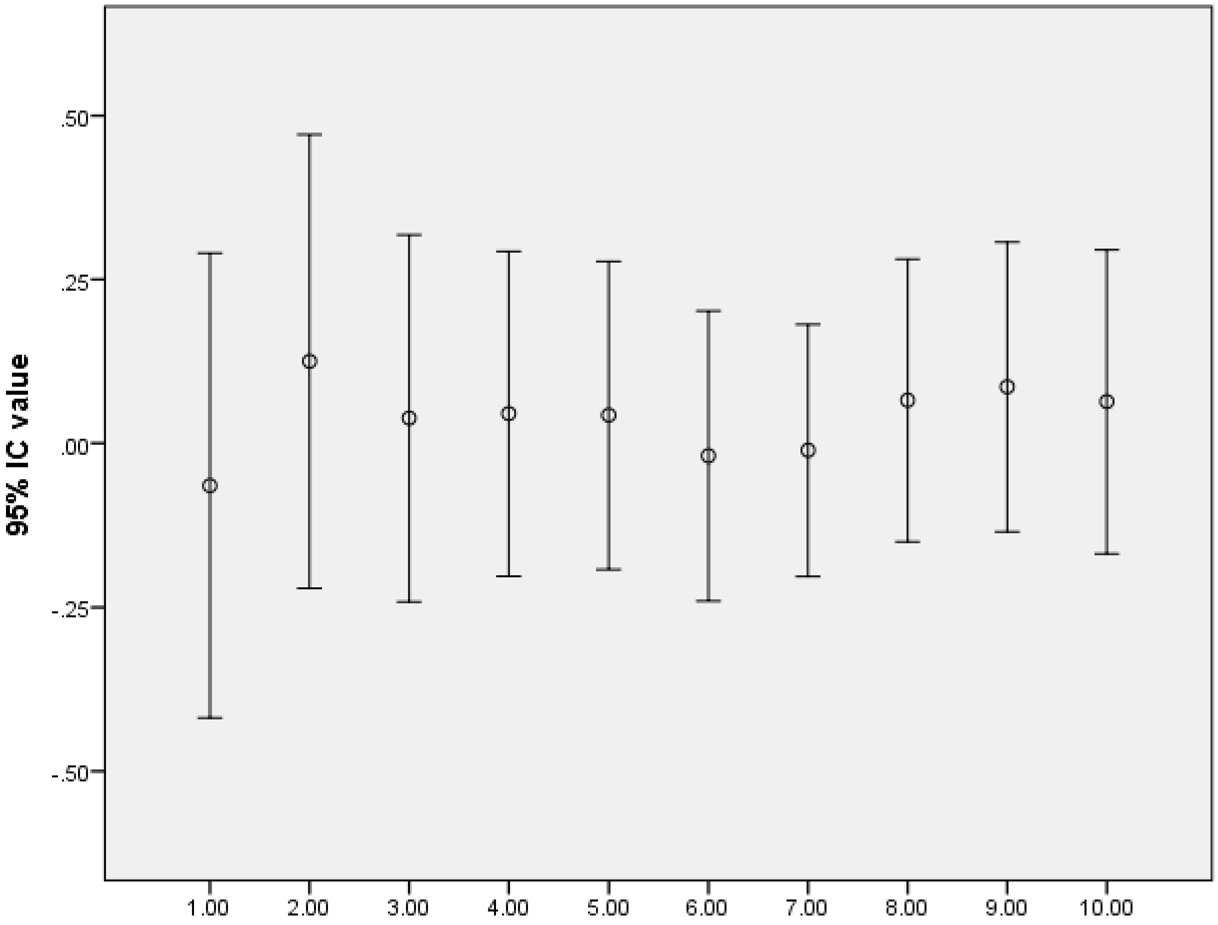

Supplement: Supplementary file 1 — Supplementary Information [file 42003_2018_125_MOESM1_ESM.pdf]
