# Supplementary material for: Ribcage measurements indicate greater lung capacity in Neanderthals and Lower Pleistocene hominins compared to modern humans
Source: Commun Biol. 2018 Aug 16;1:117. doi: 10.1038/s42003-018-0125-4 (PMC6123625; doi:10.1038/s42003-018-0125-4)
Supplement: Supplementary file 2 — Description of Additional Supplementary Information [file 42003_2018_125_MOESM2_ESM.docx]

**Description of Additional Supplementary Files**

File Name: Supplementary Data 1

Description: ID, sex, TLC (l), stature (cm) and lean body mass (kg) of the individuals studied. Stature estimations for fossil specimens were taken from Churchill9 and lean body mass estimations were calculated using total body mass values provided by Churchill9 but applying fat-free mass from Snodgrass and Leonard51. TLC/S and TLC/M are also shown. TLC, TLC/S and TLC/M values for Kebara 2 and Tabun 1 represent means calculated from different ribs (see material section) but for the case of ATD6 hominins we show both estimations, since we do not have certainty that the ribs used here (ADT6-89+206 and ADT6-39) belong to the same individual. TLC/S and TLC/M could not be calculated in the El Sidrón specimen because of stature and weight were unknown for that individual.

File Name: Supplementary Data 2

Description: raw measurements of TVA_sml of every rib level of the individuals studied as comparative sample, as well as their TLC values. The measurements of fossil ribs are also shown.
